# Hospital Administration

**Published:** 1896-04-25

**Authors:** 


### HOSPITAL ADMINISTRATION.

### THE SOUTHPORT INFIRMARY.

A special meeting of the governors of the Southport Infirmary was held on April 8th to consider an amended set of rules proposed by the committee. Two of these rules are, we think, open to some objections. Rule 4 vests the presidency of the institution in the mayor of Southport for the time being. Now, it is undoubtedly very desirable that those who have civic influence should be interested in the infirmary; but it must be borne in mind that to make a really good hospital president a man should, above all things, be interested in hospitals, and it is to be feared that in making the presidency a mere *ex-officio* appanage of the mayoralty much risk will be run of the infirmary suffering, for a mayor is of necessity a busy man, and by no means necessarily interested in hospital work. The other rule to which we take exception is that in regard to the nursing institute which it is proposed to establish in connection with the infirmary, in which it is stated that any ultimate profit from the nursing institute shall be handed over to the general funds of the infirmary. This is clearly wrong. The profits earned by the nurses should pay interest on all expenses incurred in establishing the institute, and also should provide a sinking fund for the repayment of such expenses in a short term of years, but all profits beyond that should go to the nurses who have earned them.

### HOTCH-POTCH REFORMERS.

The inchoate condition of mind which animates most of those who aspire to reform the hospitals is well shown by the proceedings at a recent meeting of the London and District Poor Law Officers' Association, when Mr. Charles Wray, consulting oculist to the Croydon Guardians, in reading a paper entitled "The Future of London Hospitals," defined the hospital future to be a question of finance—a question which had begun to grow acute from the time of the conversion of Consols. Hospital income had diminished through decreased interest from Consols, and had led in some cases to investment in questionable securities. This experience, he thought, would expedite the much-needed reform in hospital management, a reform having for its object economical administration. He severely criticised the practice of building hospital accommodation for the purpose of having empty beds wherewith to appeal to the public. Great economy could also be effected in building operations, for one institution spent £30,000 in providing accommodation that another institution could get for £9,000. Of course, the explanation was that in the former cases expensive fads had been considered, such as rounded corners, no angular windows, and fireproof floors. He did not think they could look for an improvement in hospital finance by obtaining payments from patients, because these must be almost nominal, or the patients would go to private medical practitioners, and the medical men at the hospitals would require payment for their services if fees were charged. Then State aid from the rates was not to be advocated, because that would mean the destruction of voluntary support. He thought something could be done in removing hospitals to the country, setting free valuable London sites, and reducing the cost of administration. Only accommodation for accidents and urgent cases, which was, comparatively speaking, small, need be provided in the metropolis. Discussion followed, and several speakers urged the taking over of hospitals by the Charity Commissioners, a federated hospital board, co-operation to prevent imposition by patients, and a Government inquiry to redress the admitted evils of hospital administration.

